# Characterization of the complete mitochondrial genome of *Sogatella kolophon* (Hemiptera: Delphacidae)

**DOI:** 10.1080/23802359.2020.1869613

**Published:** 2021-02-08

**Authors:** Fang Yu, Zhi-Shun Song

**Affiliations:** Jiangsu Key Laboratory of Biofunctional Molecule, School of Life Sciences, Chemistry & Chemical Engineering, Jiangsu Second Normal University, Nanjing, China

**Keywords:** *Sogatella kolophon*, mitogenome, phylogeny

## Abstract

In this study, we sequenced and annotated the complete mitochondrial genome of *Sogatella kolophon* (Kirkaldy) (Hemiptera: Delphacidae). The mitogenome was 16,708 bp in length, containing 13 protein-coding genes (PCGs), 22 tRNA genes, two rRNA genes and a control region. All PCGs started with ATN, except for *atp6* and *nad5*, which used noncanonical codon GTG, respectively. All tRNAs could fold into typical clover-leaf secondary structures, with the exception of two *trnS* genes, lacking the dihydrouridine (DHU) stem.

The delphacid genus *Sogatella* (Hemiptera: Delphacidae) is a cosmopolitan insect group with some serious pests of rice in Asia and south and central America by direct feeding on the phloem tissues and as vectors of plant virus diseases (Asche and Wilson 1990). As one of the most widely distributed species, *Sogatella kolophon* (Kirkaldy 1907) is the vector of *Digitaria* striate virus (DSV), Maize sterile stunt virus (MSSV), and Brazilian wheat spike virus (BWSV) (Wilson 2005). Here, we report the complete mitochondrial DNA genome of *S*. *kolophon* to facilitate better understanding its evolution within *Sogatella*.

Adults of *S*. *kolophon* were collected in Shimen County (N 29.94° and E 110.78°), Hunan Province, China. Voucher specimen (F4-037) and its DNA were deposited in the Institute of Zoology, Chinese Academy of Sciences, Beijing, China. Genomic DNA was extracted using the DNeasy Blood & Tissue Kit (Qiagen, Hilden, Germany) following the manufacturer’s protocols. PCR primers for amplification of the mitogenome were modified from those used in Yu and Liang (2018). Purified PCR products or multiple clones were sequenced directly on an ABI 3730XL DNA Analyzer using BigDye v3.1 (Applied Biosystems, Waltham, MA). After being assembled, the mitogenome sequence was annotated with MitoZ v2.4-alpha (Meng et al. 2019), and the annotation of tRNAs was verified by MITOS Web Server (Bernt et al. 2013).

The complete mitochondrial genome of *Sogatella kolophon* (GenBank accession number: MW009064) was 16,708 bp in length. The mitogenome encodes the whole set of genes, including 13 protein-coding genes (PCGs), 22 tRNA genes, two rRNA genes, and a control region, as observed in most insects. Gene rearrangement was detected in the mitogenome of *S*. *kolophon*, congruent with those of *S. furcifera* and *S. vibix*, in which two gene clusters *trnW-trnC-trnY* and *trnT-trnP-nad6* undergo conversion to *trnC-trnW-trnY* and *nad6-trnP-trnT*, respectively (Zhang et al. 2014; Huang and Qin 2018).

Eleven of all PCGs initiated with the canonical start codons ATG or ATT, except for *atp6* and *nad5*, beginning with GTG, respectively. For 13 PCGs, three stop codons were used: T (*atp6*, *cox1*, *cox3*, *nad5*), TAG (*cox2*, *cytb*, *nad1*, *nad3*), and TAA (other five PCGs). All 22 typical tRNA genes were found in the *S*. *kolophon* mitogenome, ranging from 56 to 71 bp. The predicted secondary structures of all tRNA genes were typical cloverleaf with exceptions of *trnS1* (AGN) and *trnS2* (UCN), of which both lack the dihydrouridine (DHU) stem.

The *nad4l-nad4* overlap was 7 bp (ATGTTAA) in size, and not identical to that of *atp8-atp6* (GTGTTAA). There were a total of nine intergenic spacers throughout the mitogenome of *S*. *kolophon*, ranging from 1 bp to 57 bp. The intergenic spacer between *trnS2 (UCN)* and *nad1* was 17 bp in length. As the largest noncoding region, the control region spanning 2304 bp is located between *rrnS* and *trnI*. In the control region of *S*. *kolophon*, tandem repeat unit was detected, identical with the 21 bp repeat unit in that of the white-backed planthopper *Sogatella furcifera* (Zhang et al. 2014).

Each PCG was translated into amino acid sequence, and aligned with MAFFT v7.394 (Katoh and Standley 2013). The concatenated dataset of 13 PCGs was generated via FASconCAT-G v1.04 (Kück and Longo 2014). A maximum-likelihood tree (Figure 1) was reconstructed employing the site heterogeneous model PMSF (Wang et al. 2018) with 1000 replicates of ultrafast bootstrap in the IQ-TREE v1.6.10 (Nguyen et al. 2015). We used the meadow spittlebug *Philaenus spumarius* (Hemiptera: Aphrophoridae) and *Pentastiridius* sp. (Hemiptera: Cixiidae) as outgroup.

**Figure 1.**
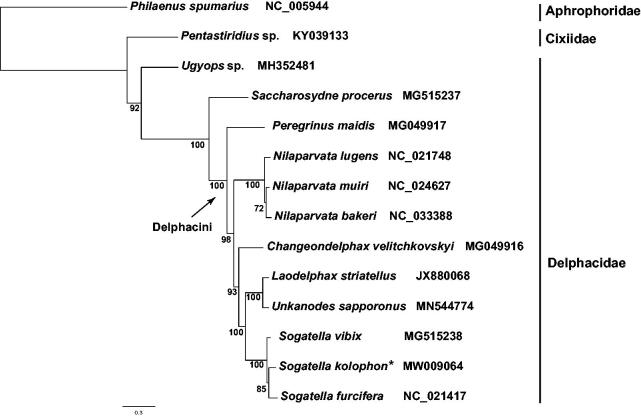
Maximum-likelihood phylogenetic tree inferred from 13 protein-coding genes. Bootstrap support values are given on nodes. Each family is indicated by vertical lines, and two species from Aphrophoridae (Hemiptera: Cercopoidea) and Cixiidae (Hemiptera: Fulgoroidea) are chosen as outgroup. *Sogatella kolophon* is marked with asterisk.

In the clade of Delphacinae, *Saccharosydne procerus*, representative of Saccharosydnini was sister group to the included species of Delphacini. *Nilaparvata lugens* was sister to *N*. *muiri*+*N*. *bakeri*. *Unkanodes sapporonus* and *Laodelphax striatellus* were clustered together, indicating their relatively closed relationships. In the genus *Sogatella*, *S*. *vibix* appeared as sister group to *S*. *kolophon* and *S*. *furcifera* with strong support.

## Data Availability

The data that support the findings of this study are openly available in figshare at http://10.6084/m9.figshare.12250331.
